# Clinicopathological characterization of chronic lymphocytic leukemia with *MYD88* mutations: L265P and non-L265P mutations are associated with different features

**DOI:** 10.1038/s41408-020-00351-w

**Published:** 2020-08-26

**Authors:** Wen Shuai, Pei Lin, Paolo Strati, Keyur P. Patel, Mark J. Routbort, Shimin Hu, Peng Wei, Joseph D. Khoury, M. James You, Sanam Loghavi, Zhenya Tang, Hong Fang, Beenu Thakral, L. Jeffrey Medeiros, Wei Wang

**Affiliations:** 1 Departments of Hematopathology, The University of Texas MD Anderson Cancer Center, Houston, TX 77030 USA; 2 Departments of Lymphoma and Myeloma, The University of Texas MD Anderson Cancer Center, Houston, TX 77030 USA; 3 Departments of Biostatistics, The University of Texas MD Anderson Cancer Center, Houston, TX 77030 USA

**Keywords:** Chronic lymphocytic leukaemia, Chronic lymphocytic leukaemia

## Abstract

*MYD88* mutations in chronic lymphocytic leukemia (CLL) are not well characterized. Earlier reports yielded conflicting results in terms of clinicopathologic presentation and prognostic impact of *MYD88* mutations in CLL patients. In addition, the morphological and immunophenotypic features of CLL cases carrying *MYD88* mutations have not been explored. Finally, the clinical or biologic implications of the canonical L265P *MYD88* mutation vs. mutations in other sites of *MYD88* within the context of CLL are also unknown. In this study, a cohort of 1779 CLL patients underwent mutational analysis, and 56 (3.1%) cases were found to have *MYD88* mutations, including 38 with L265P mutations (designated here as group A) and 18 with non-L265P mutations (group B). Cases with wild type *MYD88* were included as controls. There was no morphological difference in cases with and without *MYD88* mutations. Immunophenotypically, cases with mutated *MYD88* (both groups A and B) more frequently had an atypical immunophenotype when compared to wild type cases. Group A patients were younger and were associated with variable favorable prognostic factors, including less elevated β2-microglobulin level, negative CD38 and ZAP70, higher frequency of mutated *IGHV* and isolated del(13q14.3), and lower frequency of del(11q22.3) and mutations of *NOTCH1* and *SF3B1*. In contrast, group B patients were more similar to CLL patients with wild type *MYD88*. There was no difference in time to first treatment when comparing *MYD88*-mutated vs. wild type CLL patients before and after stratification according to *IGHV* mutation status. In summary, *MYD88* mutations are uncommon in CLL and cases with L265P mutation have distinctive clinical, immunophenotypic, cytogenetic, and molecular features. There is no significant impact of *MYD88* mutations on time to first treatment in CLL.

## Introduction

Chronic lymphocytic leukemia/small lymphocytic lymphoma (CLL/SLL) is a neoplasm of mature clonal B cells that involves blood, bone marrow, and often lymphoid tissues^1^. CLL is the most common type of leukemia of adults in the Caucasian population and has a lower incidence in Asia^2^. It represents a heterogeneous disease with a highly variable clinical course^3^. Many clinicopathological features are known to be associated with disease prognosis, such as β2-microglobulin level, immunoglobulin heavy chain variable region (*IGHV*) mutation status, ZAP-70 and CD38 expression levels, deletion 11q, deletion 17p, isolated deletion 13q, and various genetic mutations^4–12^. Mutations in different signaling pathways have been identified in CLL, some of which are associated with prognosis. For example, mutations in *TP53, ATM, NOTCH1, SF3B1*, and *BIRC3* are associated with a poorer outcome^13–15^. Myeloid differentiation primary response 88 (*MYD88*) mutations have been described uncommonly in CLL and their prognostic impact is not clearly defined.

*MYD88* is located on chromosome 3p22.2 and encodes an adapter protein that recruits interleukin 1 receptor-associated kinase 4 (IRAK4). The MYD88:IRAK4 complex recruits the IRAK4 substrate IRAK2 or the related IRAK1, forming critical signaling mediators of the Toll-like receptor (TLR)/interleukin-1 receptor (IL-1R) superfamily^16^. The *MYD88* L265P mutant is involved in spontaneously assembling the myddosome complexes, which in turn leads to NF-kB signaling activation, JAK kinase activation of STAT3, and secretion of cytokines^17^.

Mutations in *MYD88* have been studied extensively in lymphoplasmacytic lymphoma/Waldenstrom macroglobulinemia (LPL/WM) and diffuse large B-cell lymphoma (DLBCL), due to the relatively high frequency of *MYD88* mutations in these neoplasms. Greater than 90% of LPL cases have a *MYD88* L265P canonical or hotspot mutation, whereas rare cases of LPL have non-L265P mutations^18–20^. The *MYD88* L265P mutation occurs in 20–30% of non-GCB DLBCLs, and is rare or absent among DLBCLs of the GCB type^17,21^. In contrast, the *MYD88* non-L265P mutations are more evenly distributed among non-GCB and GCB DLBCLs, less than 10% in each group^17^. In terms of function, the activities of various *MYD88* mutations toward NF-kB and JAK–STAT3 signaling activation are known to differ; L265P, M232T, and S243N most strongly activate the NF-kB pathway, followed by S222R and T294P, whereas the wild type *MYD88* shows the lowest activating activity^17^. For JAK-STAT3 pathway, the L265P mutation is highly related to STAT3 phosphorylation, whereas non-L265P mutations are only modestly related^17^. The genomic profiles for L265P and non-L265P *MYD88*-mutated cases also have been shown to be different in DLBCL^22^. These findings suggest that the *MYD88* L265P mutation functions differently from non-L265P *MYD88* mutations.

The reported *MYD88* mutation rate is variable in CLL patients, ranging from 1.5 to 10%^23–28^. *MYD88* mutations have been shown to be the driver mutations that arise early in CLL development^26,29,30^. The mutation allelic frequency does not significantly change in sequential CLL samples from the same patients^31^. The outcome of CLL patients with *MYD88* mutations is highly controversial; some studies showed a favorable prognosis^23,32^ whereas others showed an unfavorable prognosis^27^, or no association with prognosis^24,28^. Thus, the prognostic role of *MYD88* in CLL/SLL is still not established. This is at least partially due to the relatively small numbers of patients (<30 cases with *MYD88* mutations in each previous cohort) as well as the combination of L265P and non-L265P mutations as a single group in previous studies.

The aim of this study is to explore the clinicopathologic features of *MYD88*-mutated CLL/SLL and to determine whether L265P vs. non-L265P *MYD88* mutations show different characteristics. We therefore collected 56 CLL cases with *MYD88* mutations, and assessed their clinical, morphologic, immunophenotypic features, fluorescence in situ hybridization (FISH), and next generation sequencing (NGS) findings and correlated with the clinical outcomes. This is the largest cohort of CLL/SLL patients with *MYD88* mutations thus far.

## Methods

### Case selection

In total, 1779 CLL patients were tested for *MYD88* mutations using an NGS-based 29-gene panel (see below, NGS analysis for details) from November 1, 2015 through February 28, 2019. For controls, we randomly selected a group of 100 CLL patients similarly tested and found to not have *MYD88* mutations during the same interval.

For the diagnosis of CLL, bone marrow aspirate smears or hematoxylin and eosin-stained tissue sections were reviewed and correlated with the results of immunohistochemical and flow cytometric immunophenotypic results. Each case was diagnosed and classified using the current World Health Organization criteria. The corresponding medical records were reviewed to obtain clinical and laboratory parameters including: age, sex, absolute lymphocyte count, serum paraprotein and β2-microglobulin levels, treatment and follow-up data. This study was approved by the Internal Review Board of our institution.

### Immunohistochemistry and flow cytometry immunophenotypic analysis

Immunohistochemical stains were performed using formalin-fixed, paraffin-embedded tissue sections either at the time of diagnosis or retrospectively for this study. Staining for ZAP-70 (dilution 1:500, Upstate Cell Signaling Systems, Lake Placid, NY) was performed as described previously^33^. Cases with nuclear staining in ≥20% of the neoplastic cells were considered positive. Nuclear staining of background non-neoplastic T-cells served as an internal control. A CD19/LEF1 (AbCam, Cambridge, MA) double immunostain was performed in a subset of cases. More details of applicability/methodology of the double immunostain were described previously^34,35^.

Flow cytometry immunophenotypic analysis was performed on cell suspensions of tissue biopsy specimens, peripheral blood (PB) or bone marrow (BM) aspirates using either a FACScanto II or FACSCalibur cytometer (Becton–Dickinson Biosciences, San Jose, CA, USA). Lymphocytes were gated for analysis using side scatter vs. forward scatter and CD45 expression vs. side scatter. The antibody panel included, but was not limited to, the following reagents: CD5, CD19, CD20, CD22, CD23, CD38, CD43, CD79b, CD200, FMC7, ZAP-70, and surface kappa and lambda light chains (Becton–Dickinson Biosciences, San Jose, CA).

### Somatic mutation status of the *IGHV* genes

Multiplex PCR amplification of *IGH* was performed using DNA extracted from patient samples and a master mix targeting the leader sequences of the variable (V) and joining (J) gene segments of *IGH*. Sequencing of the PCR products was performed using the Illumina MiSeq platform and compared with germline *IGHV* sequences using the LymphoTrack_MiSeq_IGH_2.3.1 Software (Invivoscribe, San Diego, CA). The percentage deviation of mutated bases from germline sequences were determined using IgBLAST, an immunoglobulin variable domain sequence analysis tool (NCBI, NIH). Only productive rearrangements without predicted stop codons or frameshift mutations were reported. The cutoff value utilized to determine the somatic hypermutation status was 2%.

### FISH analysis

FISH analysis was performed using formalin-fixed, paraffin-embedded tissue sections according to the manufacturer’s protocols. FISH probes used in this study included a panel of probes designed to detect deletions of 13q14.3, 17p13.1 (*TP53*), 11q22.3 (*ATM*), and trisomy 12 (Abbott Molecular Inc., Downers Grove, IL). A total of 200 interphase nuclei for each probe were analyzed. FISH analysis for CLL patients was usually performed at about the same time as NGS analysis.

### NGS analysis

Whole blood or BM samples were used for NGS analysis. *MYD88* mutations were assessed using an NGS-based 29-gene panel (EndCLL Assay V1) composed of a list of the following genes: *ATM, BIRC3, BTK, CALR, CARD11, CD79A, CD79B, CHD2, CSMD3, CXCR4, DDX3X, EZH2, FAT1, FBXW7, KLHL6, LRP1B, MAPK1, MUC2, MYD88, NOTCH1, PLCG2, PLEKHG5, POT1, SF3B1, SPEN, TGM7, TP53, XPO1* and *ZMYM3*. For *MYD88*, exon 3 (168–228), exon 4 (228–259) and exon 5 (259–310) were sequenced. In detail, 250 ng genomic DNA isolated from PB or BM was prepared for the sequencing libraries to target regions in the 29 genes. The Agilent Haloplex HS target enrichment system (Agilent Technologies, Santa Clara, CA) with molecular barcodes was utilized for library preparations. Paired-end bidirectional sequencing was performed on the Illumina MiSeq platform (Illumina Inc, San Diego, CA, USA) and a minimum average depth of 3000× and minimum 80% reads at a quality score of AQ30 or better were required. More details of the sequencing method were described previously^36^.

### Statistical analysis

Median follow-up was calculated using the inverse Kaplan–Meier method. Time to first treatment (TTFT) was calculated from the date of diagnosis to the date of first treatment. Survival distributions were calculated using the method of Kaplan and Meier, and univariate comparisons were made using the log-rank test. A subgroup survival analysis was also performed including only patients with mutated *IGHV*. Independent two-sample *t*-test and Mann–Whitney *U*-test were applied for the comparison of parametric and nonparametric continuous data, respectively. Fisher’s exact test were used for comparison of categorical variables. *P*-value ≤ 0.05 was considered as nominally significant for all analyses. The Bonferroni procedure was used to correct for multiple hypothesis testing.

## Results

### Clinical characteristics

**Fig. 1:**
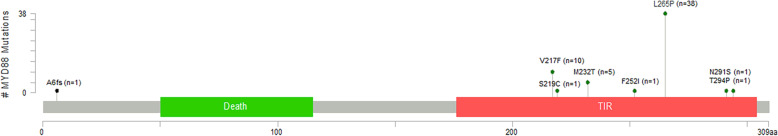
The location and frequency of *MYD88* mutations in CLL. In total, 58 mutations were identified in 56 patients. Two patients had two concurrent mutations, one with N291S/T294P, another with F252I/M232T. TIR toll/Interleukin-1 receptor.

56 of 1779 (3.1%) cases of CLL/SLL were positive for *MYD88* mutations. The canonical L265P mutation was most common, identified in 38 (68%) cases. The remaining 18 (32%) cases had non-L265P mutations, with V217F mutation in 10, M232T mutation in 4, and 1 case each of S219C, A6fs, N291S/T294P, and F252I/M232T. The distribution of *MYD88* mutations is shown in Fig. 1. For ease of discussion, we specified these cases as group A with L265P mutation and group B with non-L2565P mutations. A third group of 100 CLL patients without *MYD88* mutations (wild type; WT) served as a comparison group. The main clinical characteristics of these three groups are listed in Table 1.

**Table 1:** Comparison of clinicopathological features of 156 CLL/SLL patients with or without *MYD88* mutations.

Features		WT (*n* = 100)	A (L265P, *n* = 38)	B (Non-L265P, *n* = 18)	*P*-value		
					WT vs. A	WT vs. B	A vs. B
Age @ dx Median Range		58.5	55	61	***0.0077***	NS	***0.0071***
		(30–81)	(23–73)	(51–79)			
M:F		2.2:1 (69:31)	4.4:1 (31:7)	2:1 (12:6)	NS	NS	NS
Rai stage III–IV @ dx		14% (13/91)	5% (2/38)	6% (1/16)	NS	NS	NS
1° or 2° relative with CLL		12% (10/83)	11% (4/35)	13% (2/15)	NS	NS	NS
ALC, × 10^9^/L Median Range		16.6	13.4	22.3	NS	NS	NS
		(1.3–328.6)	(2.0–238.8)	(0.3–90.8)			
Serum Paraprotein +		9% (9/100)	11% (4/38)	6% (1/18)	NS	NS	NS
Elevated β2-microglobulin		62% (53/85)	38% (14/37)	56% (10/18)	***0.0173***	NS	NS
CD38+		47% (47/100)	16% (6/38)	17% (3/18)	***0.0008***^a^	***0.0195***	NS
ZAP-70+		68% (65/95)	23% (8/35)	53% (9/17)	***<0.0001***^a^	NS	**0.0565**
Mutated *IGHV*		39% (34/87)	97% (35/36)	63% (10/16)	***<0.0001***^a^	NS	***0.0022***

*CLL/SLL* chronic lymphocytic leukemia/small lymphocytic lymphoma, *WT* wild-type, *NS* nonsignificant, *ALC* absolute lymphocyte count. The *P*-values close to or ≤0.05 are shown in bold with values ≤0.05 in italics as well.

^a^Statistically significant after the Bonferroni correction for multiple testing (*P* ≤ 0.05/30 = 0.0017).

The median age at diagnosis for group A was 55 years (range 23–73), for group B 61 years (range 51–79), and for the WT (control) group 58.5 years (range 30–81). Group A patients were significantly younger than the WT group and group B patients (*P* = 0.0077 and 0.0071, respectively), whereas there was no age difference between group B and WT group. There was a male predominance in all three groups with a male-to-female ratio of 4.4:1, 2:1, and 2.2:1 for groups A, B, and WT, respectively. However, when limited to patients with V217F mutation from group B, females were more common (six patients) with a male-to-female ratio of 2:3.

There was no difference among the three groups in terms of Rai stage at diagnosis. The rates of first or secondary-degree relatives with CLL were similar among these three groups (Table 1). No significant difference was found in absolute lymphocyte count (ALC). Lymphocytosis was seen in 49 (88%) of CLL patients with *MYD88* mutations (group A, 34/38; group B, 15/18; *P* = NS). A paraprotein was detected in 5 (9%) patients with mutated *MYD88*; four cases in group A, including one with IgM (1.6 g/dL), two with IgG (0.4 g/dL, 2.3 g/dL) and one with both IgG and IgM paraproteins (0.3, 0.9 g/dL); and one case was in group B with IgM (0.1 g/dL). Paraproteins were detected in WT patients with a similar frequency of 9%, including six cases with IgM and three cases with IgG (range 0.1–3.5 g/dL). β2-microglobulin was elevated in 14 of 37 (38%) in group A patients, significantly less than the WT patients (53 of 85, 62%) (*P* = 0.0173). Group B had 10 of 18 (56%) patients with elevated β2-microglobulin levels, not significantly different from patients in the group A or WT groups.

### Morphologic and immunophenotypic features of CLL/SLL cases with *MYD88* mutations

**Fig. 2:**
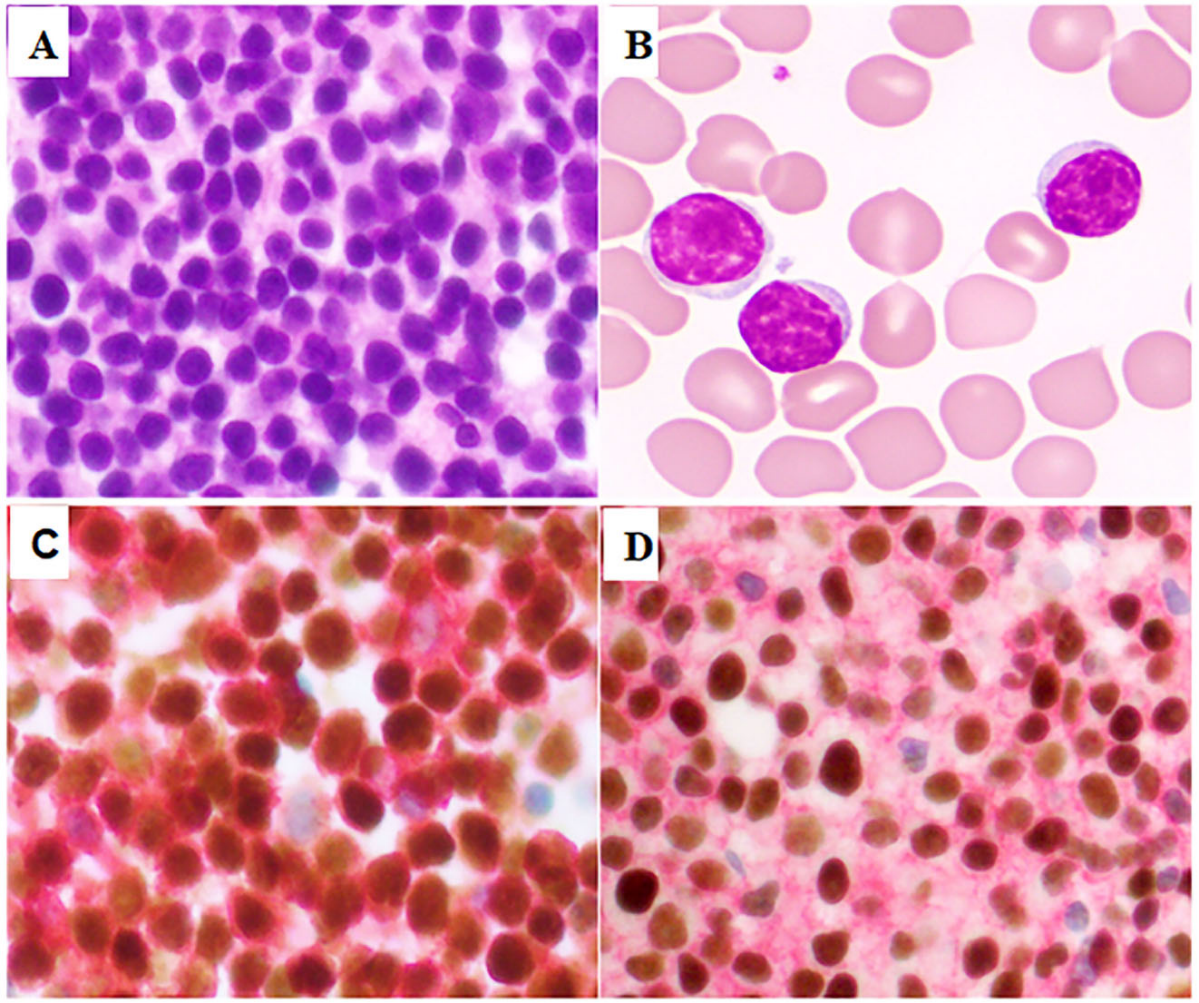
A representative case of CLL with *MYD88* mutation. **a**, **b** CLL cells are small with round to slightly irregular nuclei, condensed chromatin, and small amounts of cytoplasm. Lymphoma cells are positive for CD5 and PAX5 (**c** double stain) as well as CD19 and LEF1 (**d** double stain).

Cases with available BM aspirate smears, clot and biopsy specimens were examined and correlated with *MYD88* status. Most cases with *MYD88* mutations (group A: 85%, 23/27; group B: 71%, 10/14; *P* = NS) showed a nodular and interstitial or interstitial only pattern; eight patients (four group A and four group B; *P* = NS) showed at least focally diffuse pattern. All cases had typical CLL morphology; the cells were small with round nuclei, condensed chromatin and scant cytoplasm. Prolymphocytes were present but were few and scattered throughout the neoplasm; no cases with prolymphocytic or large cell (Richter) transformation were identified. Proliferation centers were identified in the bone marrow in a few cases (five in group A and one in group B). No CLL cases with *MYD88* mutations showed significant plasmacytic differentiation; only one case in group A showed a slightly increased plasma cell count of 4%. The morphologic and immunohistochemical presentation of a representative case from group A is illustrated in Fig. 2.

For immunophenotype, we first focused on two prognostic markers CD38 and ZAP-70 by flow cytometry. Both group A and group B patients had a lower CD38 positive rate compared to the WT group (A: 16%; B: 17%; WT: 47%; A vs. WT *P* = 0.008; B vs. WT *P* = 0.0195) (Table 1). ZAP-70 positivity was less frequent in group A than in the WT group (A: 23%; WT: 68%; A vs. WT, *P* < 0.0001). No difference was observed between group B and the WT group. There was also a trend of less ZAP-70 positive cases in group A than in group B (A: 23%, B: 53%; A vs. B, *P* = 0.0565).

**Fig. 3:**
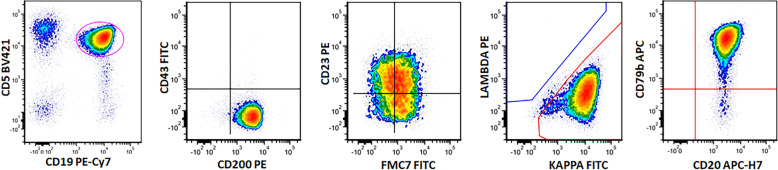
A representative case of *MYD88*-mutated CLL with an atypical immunophenotype. CLL cells were positive for CD5, CD23, and CD200, with increased expression of CD20, CD79b and surface kappa light chain. FMC7 was positive.

We next analyzed the expression of B-cell markers as well as CLL/SLL-associated markers using flow cytometry. As shown in Table 2, there were significant differences in the immunophenotype of these groups. Brighter expression of CD20, CD79b, and surface light chains were more frequently detected in *MYD88*-mutated cases than WT cases (Table 2). In addition, more cases in *MYD88*-mutated groups were positive for FMC7 and negative for CD43. There was no significant difference in CD5, CD23, or CD200 expression when *MYD88*-mutated cases were compared with WT CLL cases. The detailed percentages and statistical analysis are illustrated in Table 2, and a representative case with an atypical immunophenotype is illustrated in Fig. 3.

**Table 2:** Flow cytometry immunophenotyping of 156 CLL/SLL patients with or without *MYD88* mutations.

Flow cytometry immunophenotyping	WT (*n* = 100)	A (Hotspot *n* = 38)	B (Nonhotspot *n* = 18)	*P* value		
				WT vs. A	WT vs. B	A vs. B
CD5						
Positive	77.9% (67/86)	80.6% (29/36)	77.8% (14/18)	NS	NS	NS
Partial	22.1% (19/86)	19.4% (7/36)	22.2% (4/18)			
CD20						
Dim	95.3% (82/86)	75% (27/36)	72.2% (13/18)	***0.0021***^a^	***0.0074***	NS
Bright	4.7% (4/86)	25% (9/36)	27.8% (5/18)			
CD79b						
Dim	96.5% (83/86)	75% (27/36)	80% (12/15)	***0.0008***^a^	***0.0409***	NS
Incr	3.5% (3/86)	25% (9/36)	20% (3/15)			
K/L						
Dim	96.5% (83/86)	80.6% (29/36)	88.9% (16/18)	***0.0071***	NS	NS
Bright	3.5% (3/86)	19.4% (7/36)	11.1% (2/18)			
CD23						
Positive	81.4% (70/86)	76.5% (26/34)	83.3% (15/18)	NS	NS	NS
Partial	18.6% (16/86)	23.5% (8/34)	16.7% (3/18)			
FMC7						
Negative	87.8% (72/82)	58.1% (18/31)	55.6% (10/18)	***0.0011***^a^	***0.0036***	NS
Positive	12.2% (10/82)	41.9% (13/31)	44.4% (8/18)			
CD43						
Positive	95.2% (79/83)	73.5% (25/34)	72.2% (13/18)	***0.0007***^a^	***0.0019***^a^	NS
Negative	4.8% (4/83)	26.5% (9/34)	27.8% (5/18)			
CD200						
Positive	100% (73/73)	100% (27/27)	100% (17/17)	NS	NS	NS
Negative	0% (0/73)	0% (0/27)	0% (0/17)			

*WT* wild-type, *Incr* increased, *K* kappa, *L* lambda, *NS* nonsignificant. The *P*-values ≤ 0.05 are shown in bold and italics.

^a^Statistically significant after the Bonferroni correction for multiple testing (*P* ≤ 0.05/24 = 0.0021).

In a small subset of cases with *MYD88* mutations, CD19/LEF1 double immunostain was performed. In total, 16 cases were tested for LEF1 and all were positive in the tumor cells, confirming the diagnosis of CLL.

### Somatic mutation status of the *IGHV* genes

As shown in Table 1, 35 of 36 (97%) cases in group A were *IGHV*-mutated, which is significantly higher than that in the WT group (34/87, 39%) (*P* < 0.0001). 10 of 16 (63%) of group B patients had mutated *IGHV*, which was lower than group A (*P* = 0.0022) and not different from the WT group. In group A, the common *IGHV* gene usage was *IGHV* 3–7, 6/34 (17.6%); *IGHV* 2–5, 5/34 (14.7%); *IGHV* 3–23, 4/34 (11.8%); and *IGHV* 4–34, 3/34 (8.8%). In group B, *IGHV* 2–5 2/10 (20%) was most common, followed by one case each of *IGHV* 3–30, *IGHV* 3–73, *IGHV* 3–74, *IGHV* 3–49, *IGHV* 4, *IGHV* 4–34, *IGHV* 4–39, and *IGHV* 4–59.

### FISH and NGS analysis

**Fig. 4:**
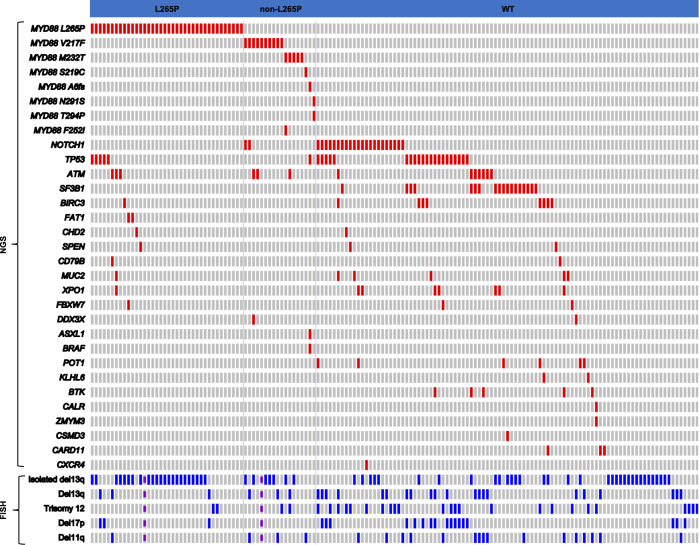
The genetic and FISH profile of CLL cases with and without *MYD88* mutations. CLL cases were divided into three groups (L265P, non-L265P, and WT). Red: NGS mutations; blue: FISH abnormalities; purple: not performed; gray: no alterations. Note: five WT cases with neither NGS mutations nor FISH abnormalities were not included in this figure.

Compared with WT *MYD88* CLL cases, group A more frequently had isolated deletion of 13q14.3 (*P* = 0.0036), and less frequently had trisomy 12 and deletion in 11q22.3 (*P* = 0.0136, and 0.041, respectively) (Table 3). In contrast, cases in group B showed no significant differences when compared to WT group. A heatmap of the major FISH findings is shown in Fig. 4.

**Table 3:** FISH and NGS analysis of CLL/SLL patients with or without *MYD88* mutations.

FISH and NGS analysis	WT (*n* = 100)	A (L265P, *n* = 38)	B (Non-L265P, *n* = 18)	*P*-value		
				WT vs. A	WT vs. B	A vs. B
FISH analysis						
Isolated del(13q14.3)	34% (34/100)	62% (23/37)	41% (7/17)	***0.0036***	NS	NS
Del(13q14.3)	55% (55/100)	70% (26/37)	59% (10/17)	NS	NS	NS
Trisomy 12	24% (24/100)	5% (2/37)	12% (2/17)	***0.0136***	NS	NS
Del(17p13.1)	17% (17/100)	8% (3/37)	0 (0/17)	NS	NS	NS
Del(11q22.3)	16% (16/100)	3% (1/37)	18% (3/17)	***0.041***	NS	0.0871
NGS analysis						
*NOTCH1* mut	22% (22/100)	0% (0/38)	11% (2/18)	***0.0005***^a^	NS	0.0994
*TP53* mut	20% (20/100)	13% (5/38)	6% (1/18)	NS	NS	NS
*SF3B1* mut	18% (18/100)	0% (0/38)	0% (0/18)	***0.0033***	0.0708	NS
*BIRC3* mut	8% (8/100)	3% (1/38)	0% (0/18)	NS	NS	NS
*ATM* mut	7% (7/100)	8% (3/38)	17% (3/18)	NS	NS	NS

*WT* wild-type, *Del or del* deletion, *mut* mutation, *NS* nonsignificant. The *P*-values ≤ 0.05 are shown in bold and italics.

^a^Statistically significant after the Bonferroni correction for multiple testing (*P* ≤ 0.05/30 = 0.0017).

As our NGS panel contains 29 genes, we also evaluated the mutation status of the remaining 28 genes listed in material and methods. Compared with the WT group, group A cases showed a lower frequency of *NOTCH1* and *SF3B1* mutations (*P* = 0.0005, and 0.0033, respectively) (Table 3). Group B cases also showed a trend toward less frequent *SF3B1* mutation compared with WT (*P* = 0.0708) (Table 3). No differences were identified in the frequencies of *TP53*, *BIRC3*, *ATM*, and other tested mutations among the three groups.

Mutations in the remaining genes of the NGS panel were uncommon. These included *XPO1* (group A, *n* = 1; WT group, *n* = 7), *CD79B* (group A, *n* = 1; WT group, *n* = 1), *FAT1* (group A, *n* = 2), *CHD2* (group A, *n* = 1; WT group, *n* = 1), *MUC2* (group A, *n* = 1; WT group, *n* = 5), *FBXW7* (group A, *n* = 1; WT group, *n* = 2), *ASXL1* (group B, *n* = 1), *BRAF* (group B, *n* = 1), *DDX3X* (group B, *n* = 1; WT group, *n* = 1), *SPEN* (group A, *n* = 1; WT group, *n* = 2), *POT1* (WT group, *n* = 6), *BTK* (WT group, *n* = 5), *CARD11* (WT group, *n* = 3), *KLHL6* (WT group, *n* = 2), *CXCR4* (WT group, *n* = 1), *CALR* (WT group, *n* = 1), *ZMYM3* (WT group, *n* = 1), and *CSMD3* (WT group, *n* = 1). These mutations did not overlap between group A and group B. A heatmap of the major NGS findings is shown in Fig. 4.

### Evaluation of patients without prior treatment

We included both untreated and previously treated patients in this study. To evaluate the potential effect of treatment, we also performed analysis using untreated patients only. As shown in the supplementary Table 1, the *MYD88* L265P-mutated cohort remained to show younger age, less ZAP70 expression, more frequent *IGHV* hypermutation, less trisomy 12 by FISH, and less *NOTCH1* and *SF3B1* mutations by NGS when compared to *MYD88* wide-type cohort. There was also a trend of less elevated β2-microglobulin and higher frequency of isolated deletion 13q in *MYD88* L265P-mutated CLL cohort. There results were similar to the analysis showed in Tables 1 and 3, which included both untreated and treated patients.

### Clinical impact of *MYD88* mutations in CLL/SLL

**Fig. 5:**
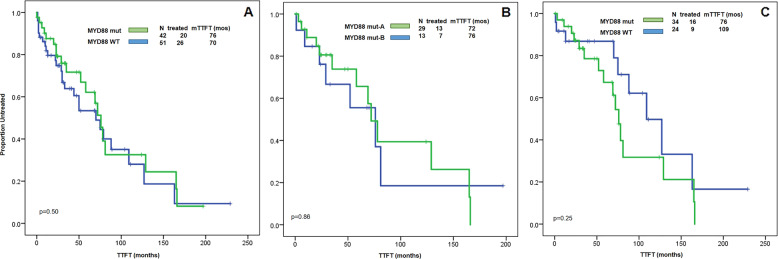
The TTFT of CLL patients with and without *MYD88* mutations. **a** Patients with WT *MYD88* vs. patients with mutated *MYD88*. **b** Patients with L265P mutation vs. patients with non-L265P mutations. **c** Only patients with mutated *IGHV* were included for TTFT analysis.

To investigate the impact of *MYD88* mutations in CLL patients, we evaluated TTFT in patients with and without *MYD88* mutations. Only patients without any prior treatment at time of *MYD88* testing were included in this analysis. There were 93 patients, 42 with *MYD88* mutations (29 in group A, 13 in group B) and 51 with WT *MYD88*. After a median follow-up of 39 months (95% CI 32-46 months), 46 (49%) patients needed to be treated, and there was no significant difference in TTFT between *MYD88* wild type and mutated groups (76 months vs. 70 months, *P* = 0.50) (Fig. 5a). There was also no difference in median TTFT when comparing group A to group B patients (72 months vs. 76 months, *P* = 0.86) (Fig. 5b). Since *IGHV* mutation status impacts survival and patients with *MYD88* L265P mutation showed a higher frequency of mutated *IGHV* (Table 1), to eliminate this confounding factor, we analyzed 58 patients with mutated *IGHV* (34 with mutated *MYD88*, 24 with WT *MYD88*). As shown in Fig. 5c, there was no significant difference in TTFT. Too few deaths (only two patients) were observed to allow statistical analysis of factors associated with overall survival (OS).

## Discussion

The overall aim of this study was to assess the clinicopathologic and prognostic features of *MYD88* mutations in CLL. In earlier reports, the relationship of *MYD88* mutations to age in CLL has been controversial. Martinez-Trillos et al studied 23 patients with Toll-like receptor (*TLR)/MYD88* mutations and found that mutated patients were significantly younger (median age, 47 years) than unmutated patients (median age, 61 years)^23^, whereas in another two studies by Baliakas et al.^28^ and Qin et al.^27^, the authors showed no age difference. Of note, all these studies lumped different *MYD88* mutations as one group. When we stratified cases into two groups based on mutation sites (i.e., L265P vs. non-L265P), we found that the L265P mutation was identified in younger patients, whereas other mutations were not (Table 1). Thus, the inconsistent findings in previous studies about age are likely attributable to combining all mutated cases together as a single group. Previous studies on the prognostic impact of *MYD88* mutations in CLL also have been discordant, as some studies showed a favorable outcome^23^ whereas others showed an unfavorable prognosis^27^, and yet others showed no prognostic significance^24,28^. In the study by Martinez-Trillos et al. the authors found that *TLR/MYD88*-mutated CLL was seen in a group of young patients with advanced stage at presentation, associated with mutated *IGHV*, with less ZAP-70 and CD38 expressions and a better OS^23^. After adjusting for the *IGHV* status, patients with *MYD88* mutations still showed a significant better OS than patients with WT *MYD88*^32^. Whereas in the study by Baliakas et al, the authors studied 21 cases of CLL with *MYD88* mutations. When focusing on *IGHV*-mutated CLL cases, patients with *MYD88* mutations showed a tendency for shorter time to first treatment (TTFT) compared with patients with WT *MYD88*^28^, and no differences were observed in terms of OS^28^. Similar results with no prognostic difference in CLL with *MYD88* mutations were described in several other studies^24,31,37^. On the contrary, in a study by Qin et al, *MYD88* mutations predicted an unfavorable prognosis in patients with mutated *IGHV* gene^27^. In our study, although L265P mutation was associated with several favorable prognostic factors, we did not find a difference in TTFT among patients with wild type vs. mutated *MYD88*. Of note, evaluating prognosis in this scenario is challenging due to the low frequency of *MYD88* mutations, the clinically indolent nature of CLL, and the presence of other confounding factors associated with prognosis.

The 3.1% mutation rate of *MYD88* gene detected in our study is similar to 3.9% detected by Martinez-Trillos et al^23^, higher than 1.5% detected by Jeromin et al.^24^ and 2% detected by Baliakas et al.^28^, and lower than 10% by Wang et al.^25^. Of note, the prior treatment, tumor burden and the assays used to detect the mutations likely contribute to the variable mutation rates reported in different studies. In addition, the prevalence of *MYD88* mutations in CLL seems to be higher in Asian population^26,27^.

*CD79B* mutation was previously shown to act synergistically with *MYD88* L256P mutation activating the NF-kB pathway in non-GCB DLBCL and was associated with improved survival in these cases^22^. In our study, *CD79B* mutation was identified only in one case in *MYD88* L265P mutation group and one case in WT *MYD88* group. Other genetic mutations associated with NF-kB pathways such as *CD79A* and *CARD11* were also very rare. The rarity of these coexisting mutations excluded the possibility to study their synergistic effect in CLL.

We did not observe preferred *IGHV* 3–23 usage in *MYD88*-mutated patients, unlike a previous report^28^. In our study, *IGHV* 3–7, not 3–23, was the most frequent gene used in L265P mutation group. A previous study showed the absence of *IGHV* 4–34 usage in *MYD88*-mutated cases^27^, whereas in our study, *IGHV* 4–34 usage was found in 9% of cases with *MYD88* L265P mutation.

As the mutation status of *MYD88* has been associated with a different response to ibrutinib therapy in patients with DLBCL and LPL^22,30^, it is of interest to study the impact of mutation on therapy in CLL patients. In our cohort, only eight patients with *MYD88* mutations were treated with ibrutinib and the response was evaluable in five patients who all showed partial response (data not shown). Thus, a definitive conclusion about the impact of *MYD88* mutation on ibrutinib treatment response cannot be drawn from this study. Future studies are needed to include more *MYD88*-mutated CLL patients treated with ibrutinib.

The detection of *MYD88* mutation in CLL, especially the L265P mutation, raises a differential diagnosis with LPL/WM. The clinical presentation, morphology, and immunophenotype separate these patient groups. In this study, all CLL/SLL cases with *MYD88* mutations showed typical CLL cytomorphology with round nuclei, condensed chromatin, and scant cytoplasm. Prolymphocytes were present and some showed proliferation centers in the bone marrow biopsy specimen, as has been described previously^38^, which is diagnostic for CLL/SLL. In contrast to plasmacytoid lymphocytes and plasma cells as the components of LPL/WM, plasma cell differentiation was extremely rare in CLL carrying *MYD88* mutations. Only one patient in our study showed slightly increased (4%) plasma cell count. Although *MYD88*-mutated CLL cases more often had an atypical immunophenotype, the CLL cases in this study were all positive for markers characteristic for CLL including CD5, CD23, and CD200 (Table 2). LPL/WM can be positive for CD5, but very uncommon^39^. Serum IgM paraprotein, required for the diagnosis of LPL/WM, was only seen in 9% of the CLL cases in this study, similarly distributed between patients with and without *MYD88* mutations. Lymphocytosis was seen in 88% of the CLL patients with *MYD88* mutations in our study, a phenomenon rarely seen in LPL/WM.

In summary, the occurrence rate of *MYD88* mutations in our study was approximately 3% and, if only L265P mutation is considered, the occurrence rate was slightly above 2%. Our study is the first to stratify *MYD88* mutations based on mutation sites (L265P vs. others) in CLL. *MYD88*-mutated CLL cases showed no distinct morphologic features. However, immunophenotypic differences were shown as *MYD88*-mutated cases (both L265P and other mutations) more frequently had an atypical immunophenotype. CLL with *MYD88* L265P mutation represented a distinct group of patients that were younger and associated with several favorable prognostic factors, including lower levels of β2-microglobulin, lower frequency of CD38 and ZAP70 expression, and higher frequency of mutated *IGHV*. CLL with *MYD88* L265P mutation was also associated with isolated deletion of 13q14.3, a lower frequency of deletion 11q22.3, and a lower frequency of *NOTCH1* and *SF3B1* mutations. In contrast, CLL with *MYD88* nonhotspot mutations showed features more similar to CLL with WT *MYD88*. Neither L265P nor non-L265P *MYD88* mutations showed an impact on time to first treatment. Of note, the cohort in this study had a relatively short follow-up interval and therefore the impact of *MYD88* mutations on overall survival could not be evaluated.

## Supplementary information

Untreated cases
