# Supplementary material for: Clinicopathological characterization of chronic lymphocytic leukemia with MYD88 mutations: L265P and non-L265P mutations are associated with different features
Source: Blood Cancer J. 2020 Aug 26;10(8):86. doi: 10.1038/s41408-020-00351-w (PMC7450076; doi:10.1038/s41408-020-00351-w)
Supplement: Supplementary file 1 — Untreated cases [file 41408_2020_351_MOESM1_ESM.docx]

Supp Table 1. Comparison of CLL/SLL with or without *MYD88* mutations in untreated patients

| Features | | WT  (n = 51) | A  (L265P,  n = 29) | B  (Non-L265P,  n = 13) | P value | | |
| --- | --- | --- | --- | --- | --- | --- | --- |
|  |  |  |  |  | WT vs A | WT vs B | A vs B |
| **Age**  **@ dx** | **Median** | 60  (34-81) | 57  (23-73) | 60  (51-75) | ***0.0278*** | NS | NS |
|  | **Range** |  |  |  |  |  |  |
| M:F | | 2.2:1  (35:16) | 3.8:1  (23:6) | 2.3:1  (9:4) | NS | NS | NS |
| Rai stage  III-IV @ dx | | 8%  (4/50) | 3%  (1/29) | 8%  (1/13) | NS | NS | NS |
| 1° or 2° relative  with CLL | | 15%  (6/40) | 7%  (2/27) | 17%  (2/12) | NS | NS | NS |
| ALC,  x 10^9^/L | Median | 13.7  (1.3-328.6) | 11.5  (2.0-123.9) | 23.8  (4.4-90.8) | NS | NS | 0.0670 |
|  | Range |  |  |  |  |  |  |
| Serum  Paraprotein + | | 8%  (4/50) | 15%  (4/27) | 0%  (0/13) | NS | NS | NS |
| Elevated  β2-microglobulin | | 55%  (28/51) | 32%  (9/28) | 46%  (6/13) | 0.0627 | NS | NS |
| CD38 + | | 33%  (17/51) | 17%  (5/29) | 15%  (2/13) | NS | NS | NS |
| **ZAP-70 +** | | 63%  (31/49) | 26%  (7/27) | 54%  (7/13) | ***0.0036*** | NS | NS |
| **Mutated*IGHV*** | | 50%  (24/48) | 96%  (26/27) | 73%  (8/11) | ***<0.0001*** | NS | 0.0648 |
| FISH analysis | |  |  |  |  |  |  |
| Isolated  del(13q14.3) | | 41%  (21/51) | 64%  (18/28) | 50%  (6/12) | 0.0619 | NS | NS |
| Del(13q14.3) | | 53%  (27/51) | 71%  (20/28) | 67%  (8/12) | NS | NS | NS |
| **Trisomy 12** | | 25%  (13/51) | 4%  (1/28) | 8%  (1/12) | ***0.0148*** | NS | NS |
| Del(17p13.1) | | 10%  (5/51) | 7%  (2/28) | 0  (0/12) | NS | NS | NS |
| Del(11q22.3) | | 10%  (5/51) | 4%  (1/28) | 17%  (2/12) | NS | NS | NS |
| NGS analysis | |  |  |  |  |  |  |
| *NOTCH1* mut | | 16%  (8/51) | 0%  (0/29) | 8%  (1/13) | ***0.0458*** | NS | NS |
| *TP53* mut | | 8%  (4/51) | 10%  (3/29) | 0%  (0/13) | NS | NS | NS |
| *SF3B1* mut | | 18%  (9/51) | 0%  (0/29) | 0%  (0/13) | ***0.0227*** | NS | NS |
| *BIRC3* mut | | 10%  (5/51) | 3%  (1/29) | 0%  (0/13) | NS | NS | NS |
| *ATM* mut | | 4%  (2/51) | 7%  (2/29) | 15%  (2/13) | NS | NS | NS |

WT: wild-type; Del or del: deletion; mut: mutation; NS: not significant
